# Autoimmune Thyroiditis Unveiled: A Case Report of Managing Virally Induced Symptoms in the Emergency Department

**DOI:** 10.7759/cureus.77121

**Published:** 2025-01-08

**Authors:** Ever G Roa, Jose L Rincon, Paul Dominici, Andre W Roman, Luis A Pantoja

**Affiliations:** 1 Emergency Medicine, Doctors Hospital at Renaissance, Edinburg, USA; 2 Family Medicine, Doctors Hospital at Renaissance, Edinburg, USA

**Keywords:** acute infectious thyroiditis, de quervain thyroiditis, levo-thyroxine, severe sepsis, systemic steroids, hashimoto’s thyroiditis

## Abstract

Thyroid storm is a complex medical diagnosis that, due to its lower incidence in general, can be challenging to assess if clinicians do not include it in their differential diagnosis. If diagnosed late, it can trigger life-threatening cardiac arrhythmia and multi-organ failure, leading to death. Here, we report the case of a 40-year-old female patient with an initial complaint of a sore throat. After being treated for a possible bacterial infection, it was then found that she had abnormal thyroid levels and a viral infection. This paper will describe the findings, medical decisions, prognosis, and different diagnoses considered when the patient was assessed in the emergency department and during her hospital stay.

## Introduction

Thyroid storm is a rare and life-threatening condition characterized by severe or exaggerated clinical manifestations of thyrotoxicosis. Patients with longstanding untreated hyperthyroidism (e.g., Graves’ disease, toxic multinodular goiter, and solitary toxic adenoma), acute events such as thyroid or nonthyroidal surgery, trauma, infection, an acute iodine load (including amiodarone use), or parturition can increase the risk of thyroid storm [1]. Clinical findings include tachycardia exceeding 140 beats/minute (bpm) and congestive heart failure. Hypotension, cardiac arrhythmia, and death from cardiovascular collapse also may occur. In one series of 28 cases, cardiac manifestations were predominant, with >60% of patients having severe tachycardia or atrial fibrillation. Hyperpyrexia to 104-106°F is common in thyroid storms, as is agitation, anxiety, delirium, psychosis, stupor, and coma. Many consider these features essential to the diagnosis of a thyroid storm. The mortality rate of this condition is substantial, ranging from 10% to 30%. 

We present the case of a 40-year-old female patient who arrived at the emergency department (ED) with tachycardia, hypertension, fever, neck pain, a newly diagnosed viral infection, which started one week prior, and abnormal thyroid levels. 

## Case presentation

A 40-year-old female patient with a past medical history of polycystic ovary syndrome, abnormal uterine bleeding status post hysterectomy, right-sided oophorectomy, and obesity came to the ED for evaluation of throat and neck pain. That same day, her primary care provider (PCP) told her that her thyroid panel was abnormal. The patient had been experiencing neck pain associated with tenderness to palpation for the past seven to 10 days. Therefore, she saw her PCP, who swabbed her for infection and ultimately prescribed Augmentin. Her PCP later requested thyroid labs and found them to be “abnormal FT4/FT3 (~1-2x ULN).” The patient reported rapid heartbeat, anxiety, tremors, diaphoresis, and fever, and she denied chest pain, shortness of breath, diarrhea, or other signs or symptoms at the time of the interview.

In the ED, the patient presented as febrile with a temperature of 103°F, tachycardia with a heart rate of 151 bpm, sinus tachycardia on electrocardiogram, and hypertensive (150/102). She was given a dose of acetaminophen and a 1500-mL bolus of IV lactated Ringer's, and her heart rate improved significantly. At that time, her vitals showed a pulse of 79 bpm, blood pressure of 109/68, and oxygen saturation of 96% on room air. Laboratory tests revealed a white blood cell count of 16200 th/uL with a left shift and an international normalized ratio of 1.22. Urinalysis was essentially negative. Her chemistry panel showed the following: sodium=132 mmol/L, creatinine=0.6 mg/dL, thyroid-stimulating hormone (TSH)=0.01 mU/L, free T3=6.9 pg/dL, and free T4=3.2 ng/dL. Her respiratory viral panel was positive for human Metapneumovirus; laboratory trends are seen in Table 1.

**Table 1:** Laboratory trends from arrival to final emergency department visit. TSH: thyroid-stimulating hormone; T: thyroxine; WBC: white blood cells; Sed rate: sedimentation rate

Laboratory results by date	Reference values	1/25/2024	02/01/2024	02/23/2024	04/26/2024	06/26/2024
TSH mlU/L	0.5 – 5.0 mlU/L	0.01	0.01	0.02	14.90	10.30
Free T3 pg/dL	0.8 – 2 pg/dL	6.9	4.9	2.2	2.6	2.8
Thyroid peroxidase IU/mL	<35 IU/mL			5.5		
T uptake TBI	0.8 - 1.3		0.6		0.6	
Free T4 ng/dL	0.7 – 1.9 ng/dL	3.2	3.2	0.8	1.0	1.4
Thyroid stimulating immunoglobulin IU/mL	<55 IU/mL	348		364		
T7 calculation	1.4 – 3.8		22.6	22.6		
C-reactive protein mg/dL	<0.3 mg/dL	5.9		8.8		
Sed rate mm/h	<20 mm/hr	59	73			17
Lactic acid mmol/L	<2 mmol/L	1.5				1.0
WBC th/uL	4.5 – 11 th/uL	16200	18000	9300	15200	12700

The patient’s Burch-Wartofsky score was elevated to 60 points. However, it improved significantly with fluid resuscitation.

The patient was admitted to the hospital, where she was started on methimazole 5 mg orally three times a day, prednisone 40 mg daily, and metoprolol succinate 25 mg daily, as part of the integrated workup by hospitalists and endocrinologists. 

She was also provided sequence compressive devices and Lovenox for deep venous thrombus prophylaxis.

**Figure 1:**
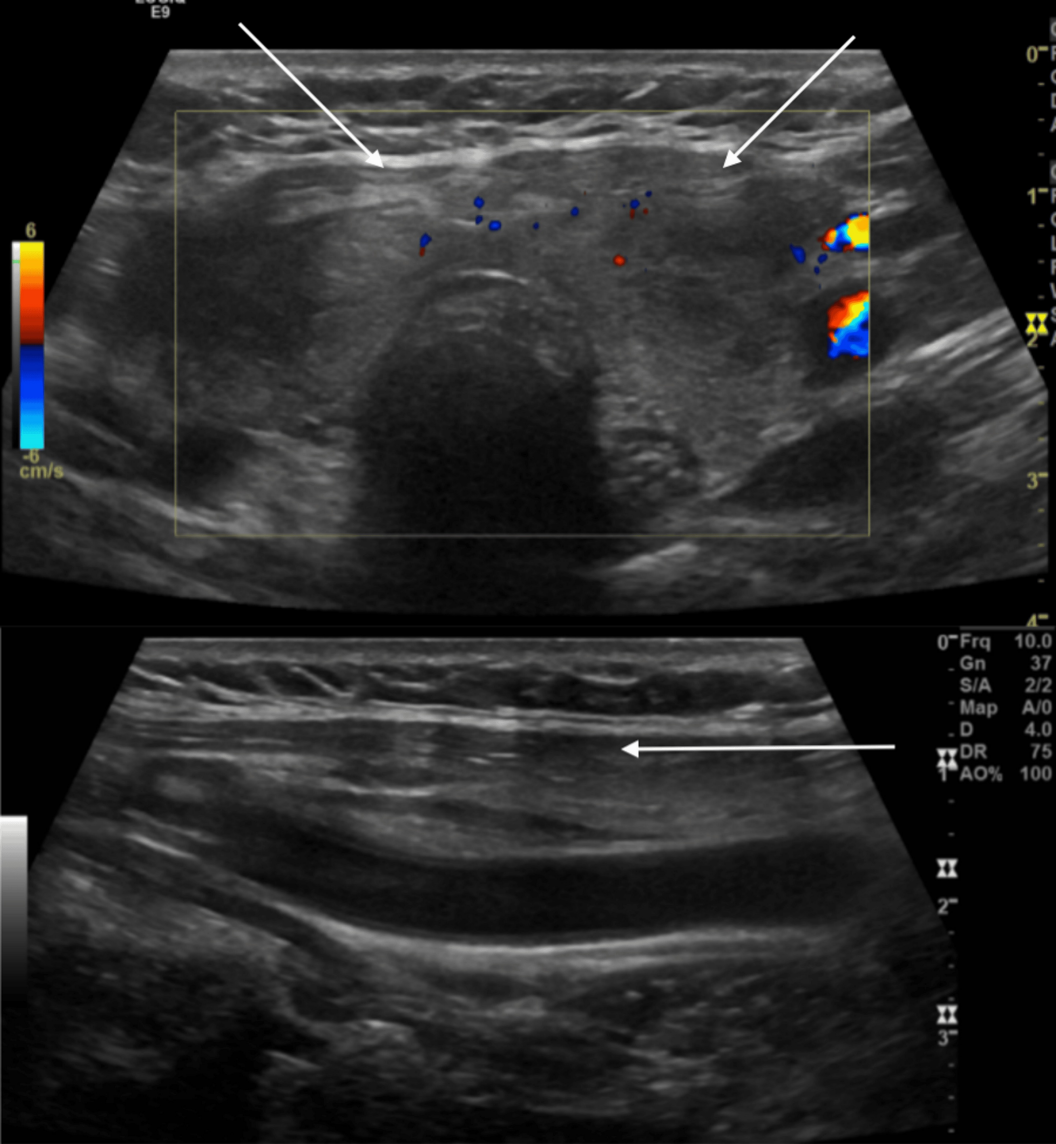
Thyroid ultrasound indicated an enlarged thyroid gland showing heterogeneous echotexture with normal vascularity.

Thyroid ultrasound indicated an enlarged thyroid gland showing heterogeneous echotexture with normal vascularity, as seen in Figure 1.

No solid mass or cystic lesion was identified. To rule out pulmonary emboli, computed tomography angiography of the chest was performed, with negative results.

Once the diagnosis was confirmed, the patient was started with the standard protocol for treating thyroiditis/thyroid storm: glucocorticoids to reduce T4-to-T3 conversion, promote vasomotor stability, possibly reduce the autoimmune process, and treat associated probable adrenal insufficiency; metoprolol succinate to treat the cardiovascular symptoms; and methimazole due to her elevated Burch-Wartofsky score. Although methimazole use is not required according to the literature [2], the decision to administer the drug was made due to the imminent risk of thyroid storm in our case. 

During hospitalization in the telemetry unit, the patient remained hemodynamically stable with progressive improvement of her condition. With normal vital signs and thyroid function tests trending down, she was discharged 72 hours after admission and was scheduled to follow up with the endocrinologist as an outpatient.

Five days after discharge, the patient presented again to the ED due to a new onset of tachycardia, low blood pressure, fever, and jitter. She was given 1.5 L of lactate Ringer, 25 mg of metoprolol, and 500 mg of Tylenol. Her thyroid panel was retested, which was trending down. The patient improved clinically after treatment and was discharged home with a new follow-up with the endocrinologist.

On the subsequent three follow-up visits, the patient remained stable, and her thyroid panel normalized. Therefore, she was discharged from the endocrinologist’s office.

## Discussion

Subacute thyroiditis secondary to Metapneumovirus infection concerning thyroid storm

The patient initially complained of neck pain, fever, and tachycardia, with TSH=0.01 uLU/mL, high free T3 and T4 levels, and a positive respiratory viral panel for Metapneumovirus. Subacute thyroiditis, or de Quervain’s thyroiditis, was, at that point, the most likely diagnosis at our assessment at the ED. The prevalence and association of subacute thyroiditis with multiple viruses vary in the literature, but it is described that numerous families of viruses can cause this condition [2,3]. Recently, the prevalence of subacute thyroiditis has been linked to COVID-19 [4,5,6]. In contrast, historically, other viral infections [7], such as enterovirus, mumps, measles, and adenovirus [2], have been the most probable causes of the disease; however, there are no documented cases triggered by Metapneumovirus. Moreover, as our patient had a Burch-Wartofsky score of 60 points, thyroid storm was a major concern, which most likely would have resulted in a medical emergency. 

As multiple authors have described [2,3,8], subacute thyroiditis is strongly associated with human leukocyte antigen (HLA)-B35 in many ethnic groups. Our patient had symptoms for two to seven weeks and had recently visited her PCP due to upper respiratory infection (URI) symptoms. Metapneumovirus is not a typical virus found in a patient with subacute thyroiditis; however, as it can be considered a URI, it would be interesting to study its association with thyroid storm further and determine whether it can be viewed as another trigger for this condition.

Treatment for subacute thyroiditis is usually conservative, involving only beta blockers, glucocorticoids, intravenous (IV) fluids, and pain medication. In this case, after thoughtful consideration, the patient was started on methimazole due to concerns of thyroid storm. 

Autoimmune thyroid disease secondary to Metapneumovirus infection 

Viral infections may contribute to the development of autoimmune thyroid diseases. In our case report, we observed a patient with multiple nodules on the thyroid gland. Previous research has shown that these nodules can lead to subclinical or persistent thyroid conditions at any age [9]. Additionally, studies by Yi K and Tian suggest that the presence of these nodules could increase the risk of developing either Graves’ disease or Hashimoto’s disease [6]. This finding could change the initial diagnosis, indicating that instead of just temporary treatment for symptoms, a longer-term approach may be needed to manage thyroid hormone levels effectively.

Sepsis secondary to thyroid abscess

In our case, the possibility of sepsis secondary to a thyroid abscess had to be considered despite the absence of an apparent thyroid mass. The patient’s presentation, characterized by fever, leukocytosis, tachycardia, and neck pain with palpation tenderness, fulfilled the systemic inflammatory response syndrome criteria even before her thyroid panel results were available, necessitating investigation for infection.

Although thyroid abscess is uncommon [9], our patient’s clinical course warranted its consideration, especially given the administration of Augmentin for a presumptive sore throat and a positive test result for Metapneumovirus infection. These findings raised concern for sepsis secondary to a potential thyroid abscess.

However, subsequent assessment, including ultrasound and CT scan of the neck, yielded negative results for thyroid masses or abscesses, effectively ruling out this differential diagnosis.

## Conclusions

In conclusion, this case of a 40-year-old woman with a complex medical history highlights the intricate interplay between viral infection and autoimmune thyroiditis, potentially resulting in a thyroid storm. The patient’s presentation in the ED with fever, tachycardia, and thyroid abnormalities necessitated prompt intervention to stabilize her condition. Our integrated approach involving fluid resuscitation, pharmacotherapy, and diagnostic imaging helped to manage the acute phase of thyroid storm effectively. Subsequent hospitalization and outpatient follow-up facilitated further monitoring and adjustment of treatment, leading to the patient’s eventual stabilization and recovery. This case underscores the importance of promptly recognizing and addressing thyroid dysfunction, especially in concurrent viral illness, to prevent potentially life-threatening complications. Continued vigilance and multidisciplinary management are crucial to ensuring optimal outcomes for patients with autoimmune thyroiditis. Clinicians must remember this condition, as it can be easily mistaken for an infection, resulting in completely different treatments and potentially harming the patient.
